# Prior immunity helps to explain wave-like behaviour of pandemic influenza in 1918-9

**DOI:** 10.1186/1471-2334-10-128

**Published:** 2010-05-25

**Authors:** John D Mathews, Emma S McBryde, Jodie McVernon, Paul K Pallaghy, James M McCaw

**Affiliations:** 1 Vaccine and Immunisation Research Group, Melbourne School of Population Health, University of Melbourne, 3010, Victoria, Australia; 2 Victorian Infectious Disease Service, Royal Melbourne Hospital, 3050, Victoria, Australia

## Abstract

**Background:**

The ecology of influenza may be more complex than is usually assumed. For example, despite multiple waves in the influenza pandemic of 1918-19, many people in urban locations were apparently unaffected. Were they unexposed, or protected by pre-existing cross-immunity in the first wave, by acquired immunity in later waves, or were their infections asymptomatic?

**Methods:**

We modelled all these possibilities to estimate parameters to best explain patterns of repeat attacks in 24,706 individuals potentially exposed to summer, autumn and winter waves in 12 English populations during the 1918-9 pandemic.

**Results:**

Before the summer wave, we estimated that only 52% of persons (95% credibility estimates 41-66%) were susceptible, with the remainder protected by prior immunity. Most people were exposed, as virus transmissibility was high with R_0 _credibility estimates of 3.10-6.74. Because of prior immunity, estimates of effective R at the start of the summer wave were lower at 1.57-3.96. Only 25-66% of exposed and susceptible persons reported symptoms. After each wave, 33-65% of protected persons became susceptible again before the next wave through waning immunity or antigenic drift. Estimated rates of prior immunity were less in younger populations (19-59%) than in adult populations (38-66%), and tended to lapse more frequently in the young (49-92%) than in adults (34-76%).

**Conclusions:**

Our model for pandemic influenza in 1918-9 suggests that pre-existing immune protection, presumably induced by prior exposure to seasonal influenza, may have limited the pandemic attack-rate in urban populations, while the waning of that protection likely contributed to recurrence of pandemic waves in exposed cities. In contrast, in isolated populations, pandemic attack rates in 1918-9 were much higher than in cities, presumably because prior immunity was less in populations with infrequent prior exposure to seasonal influenza. Although these conclusions cannot be verified by direct measurements of historical immune mechanisms, our modelling inferences from 1918-9 suggest that the spread of the influenza A (H1N1) 2009 pandemic has also been limited by immunity from prior exposure to seasonal influenza. Components of that immunity, which are measurable, may be short-lived, and not necessarily correlated with levels of HI antibody.

## Background

Lessons from past influenza pandemics, including the great pandemic of 1918-19 [1,2] can enhance understanding of later pandemics [3-5], such as the recent pandemic of H1N1 2009 [4,6-11]. Indeed, part of the genetic sequence of the H1N1 virus [12] from the 1918-19 influenza pandemic lives on in H1N1 2009 [9]. Further insights from immunology [8,13,14], from animal studies using reconstituted viruses [15], and from epidemiological analyses and modelling of past and current outbreaks and future scenarios [3,16-27] will add to understanding.

The 1918-9 pandemic was characterised by high mortality, particularly in isolated or disadvantaged populations [1,13,28]. In urban populations, mortality rates were relatively greater amongst young adults [1,2,16,17], probably because older adults were protected by persistent immunity from a related virus that had disappeared by 1890 [4,5,17,18], while children were protected by innate immune mechanisms such as those mediated by interferon [17]. The out-of-season onset in 1918 [1-3,19] likely reflected greater population susceptibility to that novel H1N1 virus, while the multiple waves of infection could have been due to waning of short-term immunity [3,5,21], antigenic drift of the virus [3,5,19,21], social distancing [22], &/or seasonal effects [5,19,29].

The pandemic of 1918-19 killed at least 0.2% of persons in most affected populations, and as many as 20% in some areas [1,2,16]. Some of this variation between populations in 1918-19 mortality has been explained by poverty [16], possibly mediated via overcrowding and malnutrition [5,16]. The very high attack-rates and mortality rates in places such as Alaska and Western Samoa [1,2,17] and amongst Aborigines in remote Australia [30] in 1918-19, and on the isolated island of Tristan da Cunha in 1971 [3,31], have led to suggestions that such isolated populations were vulnerable because they had escaped regular infection with seasonal influenza viruses and were thus left with little or no immune protection against the pandemic virus [3,5,13,17,31]. In more urbanised communities, pandemic behaviour was unusual in other ways. For example, separate waves of influenza were clearly demarcated in summer, autumn and winter in 1918-19 in England and Wales [1,2,13]. Despite those three waves of potential exposure, many persons did not report symptoms of influenza in any wave [1,2,13] (see Table 1). It has not been clear whether such persons were unexposed, whether infections were asymptomatic or unreported, or whether persons were protected by innate immunity, or by residual cross-immunity from other influenza viruses circulating before 1918 [2,3,5,17,18].

**Table 1:** Populations and observed proportions affected in summer, autumn & winter waves in 1918-19 pandemic

Population	N	- - -	S- -	-A-	- -W	SA-	S-W	-AW	SAW
South Shields	462	844.2	26.0	51.9	67.1	2.2	2.2	6.5	0

Leicester	4619	719.9	62.1	131.8	69.9	3.0	4.8	8.0	0.4

Wigan	1075	774.0	40.9	73.5	108.8	0	0	1.9	0.9

Newcastle	4461	814.4	52.5	46.6	73.1	0.4	8.7	3.8	0.4

Manchester	4686	747.1	130.8	83.7	15.6	14.3	5.5	2.3	0.6

Blackburn	1284	785.0	75.5	56.1	64.6	5.5	4.7	7.8	0.8

Widnes	3417	696.5	113.5	77.8	99.5	4.1	6.1	2.3	0

London police	746	749.3	61.7	144.8	32.2	5.4	0	6.7	0

Cambridge Uni	1766	457.0	206.7	208.4	73.6	18.7	9.6	21.5	4.5

Clifton College	451	232.8	153.0	84.3	188.5	20.0	157.4	135.3	28.8

Haileybury	515	302.9	227.2	93.2	205.8	60.2	42.7	48.5	19.4

Finchley School	1224	550.7	90.7	312.9	23.7	14.7	4.1	3.3	0

*ALL*	24706	703.1	96.5	105.0	67.5	8.1	9.3	8.9	1.6

N = total number of persons surveyed in each population. - - -: proportion of persons (per 1000) not reporting symptoms in any wave; S - -: proportion reporting symptoms in summer wave only; SA-: proportion of persons reporting symptoms in the summer and autumn waves, but not in the winter wave. SAW: proportion reporting symptoms in all three waves, etc.

Until recent work [3,32], based on data from 1918-19, there was even uncertainty about whether an attack of influenza in an early wave of the pandemic protected an individual in a later wave, as would be expected if the viruses in each wave were similar. Indeed, the English data in Table 1[2] were puzzling even to FM Burnet, the leading Australian virologist, when he and Clark reviewed the 1918-19 pandemic evidence in 1942 [13].

It occurred to us that if there were immunity in some persons before the summer wave [3,5,13,17,24], &/or if some infections were asymptomatic [3,20], this could explain the inconsistent evidence for the attack-rate in a later wave being reduced in individuals reporting symptoms in an earlier wave (Table 2). Accordingly, we now report our innovative modelling to test that hypothesis.

**Table 2:** Observed odds ratios (OR) and 95% confidence intervals to test for evidence of immune protection from wave to wave.

POPULATION	OR for SA	OR for SW	OR for AW
South Shields	1.199 (0.045 - 11.364)	0.937 (0.036 - 8.735)	1.508 (0.292 - 6.549)

Leicester	**0.292** (0.158 - 0.533)	0.871 (0.521 - 1.446)	**0.655** (0.437 - 0.979)

Wigan	0.266 (0.011 - 2.186)	0.174 (0.007 - 1.419)	0.284 (0.061 - 1.090)

Newcastle	**0.258** (0.071 - 0.816)	1.945 (1.284 - 2.936)	0.959 (0.536 - 1.692)

Manchester	0.972 (0.709 - 1.329)	1.976 (1.182 - 3.286)	1.267 (0.635 - 2.481)

Blackburn	1.033 (0.406 - 2.522)	0.782 (0.290 - 1.996)	1.729 (0.763 - 3.821)

Widnes	**0.340** (0.175 - 0.648)	**0.397** (0.230 - 0.677)	**0.219** (0.091 - 0.505)

London police	0.449 (0.117 - 1.515)	0.000 (0.029 - 2.933)	1.125 (0.324 - 3.610)

Cambridge Uni	**0.248** (0.165 - 0.371)	**0.439** (0.261 - 0.734)	0.915 (0.604 - 1.380)

Clifton College	0.302 (0.164 - 0.551)	1.055 (0.657 - 1.687)	1.756 (1.044 - 2.946)

Haileybury	1.059 (0.634 - 1.766)	**0.337** (0.199 - 0.567)	0.945 (0.550 - 1.620)

Finchley School	**0.282** (0.152 - 0.518)	1.241 (0.368 - 3.841)	**0.230** (0.060 - 0.774)

***ALL***	**0.621** (0.529-0.729)	1.105 (0.946-1.291)	0.972 (0.831-1.138)

Odds ratios (OR) and confidence intervals [33] were calculated from the data of Table 1. The numbers affected in the summer (S) wave, for example, are obtained by summing totals for S- -, SA -, S-W and SAW. An odds-ratio for SA of less than 1 (eg 0.292 for Leicester) shows a tendency for those affected in the summer wave to be less affected in the autumn wave, relative to those not affected in the summer wave. For six populations over the SA comparison, the OR (in bold type), are reduced significantly below 1, providing ostensible evidence of immune memory and protection following the summer wave. In contrast, the results of model fitting (Table 4) are consistent with effects of wave to wave immunity in all populations.

## Methods

### Data

Fourteen different sub-populations in England and Wales were surveyed in 1919. In 12 of the 14 populations each respondent was asked about symptoms of influenza during the summer, autumn, and winter waves of the pandemic [2]. Thus for each population, each person surveyed was classified into one of 8 classes according to whether they were (or were not) affected in each of the three waves. We did not use the data from Eton or Harrow schools because they did not report data for the winter wave, although the results for the first two waves were entirely consistent with the results for similar school populations over three waves. Survey results for the remaining 12 sub-populations are re-tabulated in Table 1, along with the names of the cities, institutions and schools surveyed. Younger persons predominated in a subset of four "school" populations: Haileybury and Clifton College (private boarding schools), Cambridge University, and Finchley School (a suburban school in London). Older persons (adults) predominated in the complementary subset of 8 urban populations. Because of these age and social differences, our model (below) compared parameter estimates for "school" and "urban" populations.

### Evidence of protection from wave to wave

From the data of Table 1, we re-constituted each 2 × 2 contingency table to see whether people reporting symptoms of influenza in an earlier wave were less likely, as judged by an odds ratio of less than one, to report symptoms in a later wave. The results in Table 2 show that in only 6 of 12 populations was an attack in the first (summer) wave associated with a significantly reduced risk of a repeat attack in the second (autumn) wave. There was even less evidence of protection from summer to winter and autumn to winter waves.

### Comprehensive model-fitting to 12 populations

To exploit the valuable information on repeat attacks of influenza from wave to wave, to explore the possible effects of asymptomatic infection and immunity, and to better understand the biology and transmission of influenza, we have fitted a comprehensive model to the data of Table 1.

**Figure 1:**
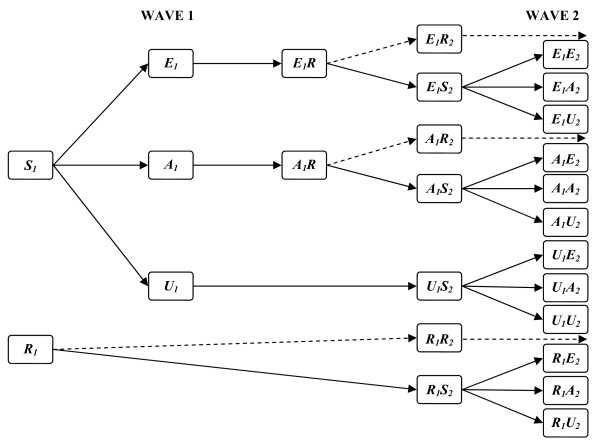
**Model for unobserved processes**. Before wave 1, people are either susceptible (*S_1_*) or resistant (*R_1_*) because of prior immunity. Persons exposed to the virus either express symptoms (*E_1_*), or have an asymptomatic infection (*A_1_*). Others are unexposed (*U_1_*). After exposure, persons become immune (*E_1_R *or *A_1_R*), and a proportion become susceptible again prior to wave 2 (*E_1_S_2_*, *A_1_S_2 _*and *R_1_S_2_*, plus the susceptible persons who escaped exposure in wave 1 (*U_1_S_2_*). All susceptible persons are at risk of exposure in wave 2 (see figure), and either express symptoms, have an asymptomatic infection, or remain unexposed. The extension to wave 3 adds an additional layer of complexity, but there are no new principles invoked.

The model allowed for the possibility of immunity before the summer wave [3,5,17,24], as well as for immune protection from one wave to the next [3,13,32]. (see Figure 1). We assumed that susceptible (ie not previously immune) persons who were exposed in a particular wave could develop symptomatic or asymptomatic infections and become immune, and we allowed for the possibility that protection could be lost because immunity waned in individuals between waves, or because of antigenic drift of the virus. Our basic model assumed homogeneous mixing within each sub-population, and we used our extension of the final size equation, an ancillary result that follows directly from the SIR model of transmission [33], to link the reported incidence of symptoms in each subgroup of the population to their susceptibility status before each wave.

### Parameter definitions

*E *= proportion of each population reporting symptoms in a particular wave.

*Z *= proportion of persons susceptible before the first wave;

Φ_1 _= proportion of non-susceptible persons (not yet infected by the pandemic virus) who become susceptible by the start of the next wave;

Φ_2 _= proportion of persons immunised by exposure to the pandemic virus who become susceptible again by the next wave;

*α_1_, α_2_, α_3 _*= proportions of immunising exposures that lead to reported symptoms in each of the three waves;

*R_0 _*= the basic reproduction number - ie the notional average number of secondary cases of symptomatic influenza from each primary case if the entire population were susceptible.

*R *= the effective reproduction number - ie the notional average number of secondary cases of symptomatic influenza from each primary case when the entire population is not susceptible. (At any time *t *during an outbreak, *R *is approximated by *R = Z(t).R_0 _*where *Z(t) *is the proportion still susceptible. Thus R declines progressively with time and *R~1 *when the epidemic peaks.)

### Estimation procedures

To measure the magnitude of each of the (unobserved) processes thought to be generating the observations, we estimated parameter values to best fit the observations using a Markov Chain Monte Carlo (MCMC) algorithm with Metropolis Hastings acceptance criterion; we introduced hyper-parameters to allow for parameter variation between populations. Full details are provided in the supplementary material in Additional File 1.

### Calculating attack rates

For each wave in each population, the predicted attack rate was calculated by iteratively solving the final size equation for *E*,

given the current values of *Z *at the start of the wave, and the other parameters. *Z *estimates were recalculated after each wave and before the next wave, in accordance with estimates for Φ_1 _& Φ_2_.

**Figure 2:**
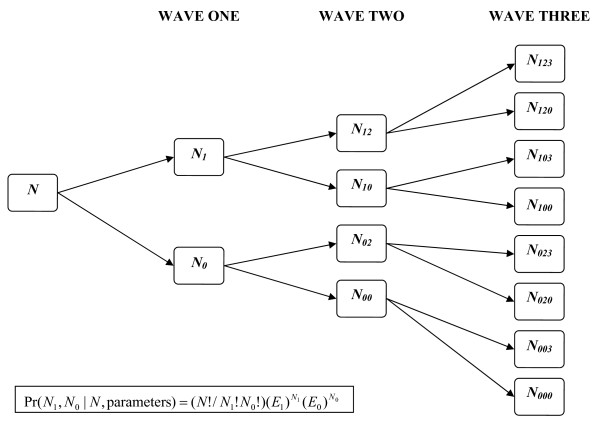
**Observations over three waves**. For each population there was information on *N *individuals, of whom *N_1 _*reported symptoms in wave 1 and *N_0 _*did not; in wave 2, *N_12 _*had a repeat attack, while *N_10 _*of those affected in wave one did not have a repeat attack; *N_123 _*individuals reported symptoms in all three waves...;;etc. The total number reporting symptoms in wave 2 was *N_12 _*+ *N_02_*, while the total number for wave three was *N_123 _*+ *N_103 _*+ *N_023 _*+ *N_003_*. The observed numbers *N_123_*, *N_120_*, *N_103_*, *N_100_*, *N_023_*, *N_020_*, *N_003_*, *N_000 _*for each population can be recovered from the proportions SAW, SA-, S-W, S- -, -AW, -A-, - -W, - - - shown in Table 1 by multiplication by the corresponding *N*.

*Likelihoods *for each sub-population were calculated from the likelihood of the parameters (given the current values of hyperparameters) and the seven conditionally independent probabilities (each determined by current parameter values), governing the sequential and parallel processes giving rise to the 2 × 2 × 2 matrix of outcomes (Figure 2). Full methods and associated references are available in Additional file 1.

## Results

### Model Results

Our biological model explained the attack-rates within each wave, as well as patterns of repeat attacks reported over the three waves of the pandemic; there was good agreement between the observed numbers and those predicted by the model (see Table 3). The parameter estimates (Table 4) were informative. Over all populations the median estimate of the proportion susceptible *(Z*) before the first (summer) wave was 52%, with a 95% credibility interval of 41-66%; this allowed us to reject the possibility that all persons were initially susceptible to the 1918 virus. Secondly, of those exposures that resulted in immunising infections, only a median proportion *α *= 39% led to reported symptoms in wave 1; the proportions in later waves were 43% and 48%. There was also a tendency for protection that antedated the first wave to lapse in a greater proportion of persons (Φ_1 _= 57%) than the protection that followed exposure to the pandemic virus (Φ_2 _= 35%), presumably because the latter protection was more specific for the new virus.

**Table 3:** Expected numbers (from model with maximum likelihood parameter estimates) and observed numbers of persons reporting symptoms in each of the three waves

POPULATION	N	SAW	SA -	S -W	S - -	-AW	- A -	- - W	- - -
**South Shields**	462	0.08 0.00	0.90 1.00	1.73 1.00	12.73 12.00	2.26 3.00	24.30 24.00	33.25 31.00	386.74 390.00

**Leicester**	4619	1.02 2.00	16.75 14.00	27.80 22.00	271.80 287.00	37.40 37.00	613.20 609.00	314.53 323.00	3336.50 3325.00

**Wigan**	1075	0.02 1.00	0.69 0.00	1.99 0.00	46.64 44.00	1.74 2.00	73.84 79.00	99.85 117.00	850.23 832.00

**Newcastle**	4461	0.41 2.00	5.26 2.00	33.22 39.00	237.09 234.00	16.55 17.00	211.94 208.00	322.86 326.00	3633.67 3633.00

**Manchester**	4686	1.84 3.00	66.94 67.00	23.49 26.00	619.65 613.00	10.49 11.00	381.88 392.00	80.22 73.00	3501.50 3501.00

**Blackburn**	1284	0.37 1.00	5.34 7.00	11.64 6.00	104.12 97.00	5.63 10.00	81.97 72.00	84.54 83.00	990.39 1008.00

**Widnes**	3417	0.40 0.00	10.66 14.00	25.40 21.00	382.55 388.00	9.84 8.00	261.65 266.00	346.08 340.00	2380.43 2380.00

**London police**	746	0.18 0.00	5.81 4.00	1.82 0.00	41.69 46.00	3.49 5.00	115.47 108.00	16.96 24.00	560.58 559.00

**Cambridge Uni**	1766	3.00 8.00	44.08 33.00	33.42 17.00	341.88 365.00	25.59 38.00	375.38 368.00	135.31 130.00	807.33 807.00

**Clifton College**	451	14.43 13.00	19.26 9.00	73.26 71.00	51.85 69.00	38.87 61.00	51.88 38.00	86.61 85.00	114.84 105.00

**Haileybury**	515	8.98 10.00	27.38 31.00	45.68 22.00	96.60 117.00	19.19 25.00	58.47 48.00	87.76 106.00	170.94 156.00

**Finchley School**	1224	0.28 0.00	18.05 18.00	2.41 5.00	111.70 111.00	5.68 4.00	372.76 383.00	26.62 29.00	686.50 674.00

***TOTAL***	24706	31.01 40.00	221.12 200.00	281.88 230.00	2318.31 2383.00	176.70 221.00	2622.77 2595.00	1634.57 1667.00	17419.64 17370.00

As before, SAW denotes the number of persons reporting symptoms in each of the summer, autumn and winter waves. S - - denotes the number with symptoms only in the summer wave ....etc. The expected numbers are based on the maximum likelihood parameter estimates for the 12 population model (simulation 1; results from simulation 2 are almost identical). The deviance corresponding to the MLE fit was 47182. It can be seen that the fit between observed and expected is less good for three of the "school" populations, reflecting their somewhat different behaviour (see also Table S2 in Additional File 1).

**Table 4:** Median parameter estimates (2.5-97.5% credibility intervals) in model to explain the observations in Table 1.

Contrast	***R***	***R_0_***	***Z***	Φ_1_	Φ_2_	α_1_	α_2_	α_3_
**All 12 ** **populations**	2.33 (1.57-3.96)	4.59 (3.10 - 6.74)	0.52 (0.41 - 0.66)	0.57 (0.42 - 0.71)	0.35 (0.23-0.46)	0.39 (0.25 - 0.52)	0.43 (0.30 - 0.59)	0.48 (0.31-0.65)

**8 urban ** **populations**	1.81 (1.16 - 2.94)	3.80 (2.53 - 5.92)	0.49 (0.34-0.62)	0.50 (0.34 - 0.76)	0.38 (0.24-0.57)	0.38 (0.20 - 0.58)	0.34 (0.21 - 0.49)	0.38 (0.22-0.59)

**4 "school" ** **populations**	2.84 (1.43-5.36)	4.73 (2.43-8.42)	0.61 (0.41-0.81)	0.74 (0.49 - 0.92)	0.38 (0.20-0.57)	0.49 (0.30-0.68)	0.64 (0.43-0.86)	0.71 (0.41-0.91)

For parameter definitions, see methods text. Estimates for *R_0_, Z, Φ_1_, Φ_2_, α_1_, α_2_, α_3 _*were derived from the joint distributions of the relevant hyperparameters; these parameters can be regarded as the typical values for populations such as those studied. By combining information from the joint distributions of *R_0 _*and *Z*, it was also possible to derive the medians and credibility intervals for the effective reproduction number *R = R_0_.Z*, at the start of the first wave. Population-specific estimates are given in Tables S.3 & S.4. For detailed methods see the supplementary information in Additional file 1. Results to support the validity of estimation procedures are given in Additional File 1 - see Tables S1 & S2. and Figure S1.

The 95% credibility intervals for *R_0 _*were estimated as 2.53-5.92 for the eight urban populations and 2.43-8.42 for the four "school" populations; the median estimates (Table 4) were similar to our earlier findings [3]. Such high values for *R_0 _*imply that if a population has negligible prior immunity, as in Alaska in 1918-19 [1,2,17], or Tristan da Cunha in 1971 [3,31], there would be a very high attack-rate. However, English populations in 1918 (Table 1) apparently had considerable prior immunity, so that the effective *R *at the start of the first wave, calculated as *Z.R_0_*, was much less (credibility interval 1.16-2.94 for urban populations & 1.43-5.36 for the "school" populations). The lower effective *R*, together with values for *α *that are considerably less than one (see Table 4), together explain why the attack rates were as low as reported (Table 1).

Comparisons of different populations provide additional insights. Attack rates were higher in partially sequestered populations in Cambridge University and private boarding schools (Clifton College and Haileybury) than in the suburban school at Finchley in London (Table 1). Parameter estimates for individual populations (see Additional File 1 Tables S3 & S4) show that higher attack rates were associated with greater susceptibility, arguably because of lesser past exposure to seasonal influenza. Furthermore, protection that antedated the first wave tended to lapse more in "schools' (Φ_1 _= 74%) than in urban populations (Φ_1 _= 50%). This is what would be expected if influenza immunity induced by past exposures increased incrementally with age, and if the rate of loss were greater when there were fewer past exposures. Further, loss of protection induced by the pandemic virus was similar for "school" (Φ_2 _= 38%) and urban populations (Φ_2 _= 38%), which would be expected following exposure to a virus that was new.

## Discussion

Our modelling results using 1918-9 data support earlier suggestions [3,17] that the spread of pandemic influenza can be limited by pre-existing immunity, probably resulting from prior exposure to seasonal influenza [34]. Furthermore, the waning of prior immunity likely contributed to the recurrent waves of influenza that characterised the 1918-9 pandemic in urban populations. These modelling inferences are necessarily tentative, as they cannot be supported by studies of immune mechanisms in those historical populations. Nevertheless, our modelling approach is innovative, biologically plausible and uses modern estimation procedures. (Additional file 1 provides further details of the methods and potential limitations of our approach.)

Our paper is able to make strong inferences about asymptomatic infections, immunity, *R *and *R_0 _*without having to estimate or guess, as is usually the case, the serial interval of influenza infection [3]. This was possible because we had data on the final size of each of the three waves in 12 sub-populations, and because we assumed homogeneous mixing within each sub-population, to underpin the (deterministic) "final-size" equation used to link the attack rate to the parameters (see methods and Additional file 1). Although the assumption of homogeneous mixing can only be an approximation, it seems reasonable, and is frequently made. Furthermore, in the supplementary information (Additional file 1) we show that our model conclusions are robust to effects arising from the simplest form of social distancing, although we cannot exclude more complex forms of social distancing as an ancillary explanation for the wave-like behaviour of influenza [22]. However, social distancing alone cannot explain why some persons had repeat attacks from wave to wave. In ongoing work, we are relaxing the assumption of homogeneous mixing, and testing the robustness of our conclusions against more complex models of social distancing.

Our results show that *R_0 _*estimates varied somewhat between populations, and tended to be greater in schools, as would be expected with higher mixing rates. In the results presented, we did not allow for systematic variation of *R_0 _*from wave to wave, as in other analyses (not shown) we found that this did not lead to a significant improvement in model fit. Our assumption of an *R_0 _*that did not vary between waves also means that we have disregarded the potential effects of seasonality on *R_0 _*and transmission [29]. However, our work suggests that the attack-rate is related more directly to the proportion susceptible *(Z)*, and to population-specific mixing as measured by *R_0_*, and that seasonal effects may be of lesser importance. Indeed, we believe that the summer onset of the first wave in England in 1918 was because the antigenic novelty of the new virus, by increasing *Z*, had over-ridden the seasonal effect.

There is precedent for our view that cross-reactive immunity induced by prior exposure to a different subtype of influenza can provide partial protection against a new pandemic strain [3,5,18,19]. Such heterosubtypic immunity is well documented in mouse models [35,36], while the evidence from human studies, although inferential, is supportive. Indeed, the very replacement of H1N1 by H2N2 in 1957, and of the latter by H3N2 in 1968 [19] provide strong circumstantial evidence for the importance of heterosubtypic immunity at the population level. Cross-immunity of short duration between different influenza strains has also been invoked to help explain the apparent constraints on the evolutionary diversification of influenza A [21]. More directly, Slepushkin reported in 1959 [37] that persons with symptoms during the H1N1 influenza in the spring of 1957 were less likely to be symptomatic in the summer when the new H2N2 influenza appeared (odds ratio = 0.418, 95% confidence interval 0.304-0.575); in the later autumn wave of H2N2, the level of protection had declined (OR = 0.625, CI = 0.530-0.737). Epstein [34] re-analysed viral isolation data from the Cleveland family study before and after the arrival of H2N2 in 1957, and found that adults known to be infected by H1N1 over the period 1950-57 were less likely to be infected with H2N2 in 1957 (OR = 0.294, CI 0.01-3.07), although the difference was not significant because of the small numbers. In the Seattle family study over the period 1975-79, coinciding with the return of H1N1 [38], the age-related decline in attack-rate was only partly explained by hemagglutination inhibition (HI) antibodies. Adults were rarely infected with H1N1 regardless of HI antibody titre, possibly because of cumulated heterosubtypic immunity from recent exposures to H3N2, although older adults in the study could have been protected by H1N1 memory from exposures prior to 1957, before H1N1 was replaced by H2N2. In 1985, Sokoguchi et al [39] reported strong cross-protection between H3N2 and H1N1 in almost contemporaneous outbreaks in Japanese schools in 1978 (OR = 0.059, CI = 0.019-0.131 for high school students, and OR = 0.154, CI = 0.076-0.309 for younger students). Such strong cross-protection when exposures to different subtypes were separated by only a few days or weeks [39] is to be contrasted with the weaker protection reported when sequential exposures were more widely separated in time [37], suggesting that at least some components of cross-protection can fade rapidly, consistent with our interpretation of the 1918-19 data.

What are the mechanisms of heterosubtypic immunity? Studies in mice and other experimental animals have implicated mucosal antibodies, CD4 and CD8 T-cells, and B cells [35,36]. Cytotoxic (CD8) T-cells reacting with conserved epitopes on internal viral proteins are of particular importance in eliminating virus-infected cells, thereby reducing the severity and duration of infection [19,34-36,40-44]. HLA-restricted CD8-mediated cytotoxic activity is also widespread in humans [36,43-45]. For example, cytotoxic cells from most healthy subjects in UK and Vietnam recognise epitopes of seasonal influenza, as well as similar epitopes of H5N1 avian influenza [45]. McMichael and others have shown that specific CD8 cells reduce viral shedding and duration of infection in people, and that cytotoxic activity fades over several years without re-exposure [43,44]. In young children, cellular immune responses induced by live-attenuated influenza vaccine appear to protect against laboratory-confirmed influenza [40].

Such collateral evidence supports our view [3,5] that in 1918-19 many people in cities were at least temporarily protected from pandemic influenza by pre-existing heterosubtypic immunity, presumably induced by recent exposure to seasonal influenza. We propose that pre-existing heterosubtypic immunity was often short-lived, and that immunity to a new strain or subtype also required several exposures before becoming more permanent. For example, it is possible that heterosubtypic protection antedating wave 1 was mediated by CD8 T-cells, which could fade over time in persons not exposed, or when exposure did not result in a significant viral load. The primary antibody response in persons exposed to larger viral loads in wave 1 could have faded in some persons before wave 2. By wave 3, immunity could have been consolidated in those with several exposures through the production of longer-lived antibody of IgG class. This more permanent protection would have helped to defer the next outbreak to the influenza season of 1920, and started the transition from pandemic to seasonal behaviour [19,46].

How do our findings relate to the 2009-10 pandemic caused by the H1N1 2009 virus of swine origin? Despite changes in social conditions since 1918, published estimates of the effective reproduction number (*R) *for the new swine flu are in the range 1.2-3.1 [47-49], with the larger estimate relating to transmission between minors in Japan [48]; these results are consistent with our findings from 1918-19, including higher rates of transmission for "schools" (Table 4). In our results we draw an important distinction between the higher estimates for *R_0 _*and the lower estimates for *R *at the start of the outbreak. This difference reflects the effect of prior immunity in moderating the spread of pandemic influenza in 1918-19 [2,3,5,24]. Unfortunately, although most influenza modellers have estimated *R*, some have reported it or used it as *R_0_*; we suggest [11] that this could systematically under-estimate [25-27] what the rate of spread of influenza would be in more fully susceptible populations, as in isolated locations such as Tristan da Cunha in 1971 [3,31] or in sequestered schools such as Saffron Walden in 1918-9 [2,3].

Although H1N1 2009 swine flu shows the pandemic signature of a relatively greater mortality in young adults [6,50], aggregate influenza mortality in 2009 [6,10] seems lower than that from seasonal influenza, which typically affects the elderly[19]. Such observations support the growing consensus that the H1N1 2009 virus has also been spreading in partially immune populations [10,11,47,51-53]. However, pre-existing cross-reactive antibodies to H1N1 2009 seem confined to older persons, presumably directed against epitopes not present in the recent H1N1 seasonal virus [8,51]. CD8+ T-cells directed against conserved influenza epitopes, which would resolve infections early, could help to explain the constrained spread of H1N1 2009 even in persons without neutralising antibody [54]. If cross-immunity is limiting the rate of spread of the H1N1 2009 virus in the same way as for the 1918-19 virus [3,5,11], and if that cross-protective immunity is also short-lived, there is a risk of repeat pandemic waves in 2010. Furthermore, it is possible that without vaccination, populations escaping early infection with the pandemic virus will experience more rapid spread or greater disease severity when eventually infected. Fortunately, trial results suggest that a single dose of pandemic vaccine can induce ostensibly protective levels of antibody, possibly by building on cross-reactive immune memory from prior exposures to seasonal H1N1 virus or vaccine [52,53].

## Conclusions

Our findings suggest that in urban populations, the spread of pandemic influenza in 1918-9 was limited by prior immunity rather than by low values of R_0_. Higher attack rates for pandemic influenza in isolated populations also reflect high values of R_0_, but with lesser levels of prior immunity, presumably because of less recent exposure to seasonal influenza. We suggest that the spread of the 2009 H1N1v pandemic may also be limited by immunity from prior exposure to seasonal influenza. Such immunity may be short-lived, and not well correlated with levels of HI antibody. It is unclear whether any recurrent pandemic waves in 2010 will have higher mortality rates, as seen in the second and third waves in 1918-19.

## Competing interests

JDM was an expert witness on the epidemiology of influenza in a matter before the Supreme Court of Victoria in 2007-8. The parties to that case have had no influence on, no involvement in, and have made no financial contribution to this work. JDM, JMcC, JMcV, and EMcB have provided advice on influenza matters at various times to the Australian Government, or to the Victorian Government.

## Authors' contributions

JDM conceived the project, wrote code, identified and analysed data, and drafted the manuscript; EMcB oversighted the Bayesian framework and hyperparameters; JMcC improved the code; all authors reviewed the ideas, methods, preliminary results, discussion and draft manuscript; all authors read and approved the final manuscript.

## Pre-publication history

The pre-publication history for this paper can be accessed here:

http://www.biomedcentral.com/1471-2334/10/128/prepub

## Supplementary Material

Additional file 1 **Figures S1.1 and S1.2. Tables S1, S2, S3, S4.**  Materials and methods. Supporting text. Social distancing. Demarcation of waves and waning of resistance. Incremental nature of acquired immunity. Possibility of virus variation between waves. Possibility of seasonal changes in bacterial infection. Comments on mortality. Click here for file
