# Supplementary material for: Prior immunity helps to explain wave-like behaviour of pandemic influenza in 1918-9
Source: BMC Infect Dis. 2010 May 25;10:128. doi: 10.1186/1471-2334-10-128 (PMC2891754; doi:10.1186/1471-2334-10-128)
Supplement: Additional file 1 — Figures S1.1 and S1.2. Tables S1, S2, S3, S4. Materials and methods. Supporting text. Social distancing. Demarcation of waves and waning of resistance. Incremental nature of acquired immunity. Possibility of virus variation between waves. Possibility of seasonal changes in bacterial infection. Comments on mortality. [file 1471-2334-10-128-S1.PDF]

Mathews Additional file 1
Supporting online information

Prior immunity helps to explain wave-like behaviour of pandemic influenza in 1918-19[1]

John D Mathews, Emma McBryde, Jodie McVernon, Paul Pallaghy,
James M McCaw

MATERIALS & METHODS

Data quality: Mortality rates were not high enough in any of the 12 populations to materially affect these incidence data [1][2]. Of greater concern would be any differential sensitivity in symptom reporting between populations. Although the clinical criteria for the diagnosis of influenza were not reported in detail for all populations, it seems reasonable to assume that the diagnostic error would be low at the time of the pandemic. For the results presented, we have implicitly taken account of reporting differences by allowing α, the proportion of those infected who reported symptoms, to vary from wave to wave (through α_1, α_2, α_3) and from population to population (via the hyperparameters). In other models (results not shown) we fitted extra parameters to estimate the sensitivity and specificity of the diagnosis of influenza, and find each to be very close to 1, with little change in the other parameter estimates.

Model details: Our biological assumptions were similar to those made previously [2]. For each of the 12 populations we assumed that a susceptible person (S_1 in Figure 1 in main paper) could be exposed and subsequently become immune. In such exposed persons, symptoms would develop (E_1) in a proportion α, whereas in a proportion (1 –

[1] This document provides supplementary information to the paper published in BMC Infectious Diseases, MS: 1643682805310893, May 2010. Enquiries to mathewsj@unimelb.ed.au

[2] These numbers in square brackets refer to supporting references at the end of this file.

α) the infection would be asymptomatic or at least not reported (A_1), and the person would become resistant (eg E_1R). Susceptibility could redevelop after infection because of waning immunity or antigenic drift (eg to E_1S_2). If the initial immunity antedated wave 1, we assumed that it could wane in a proportion Φ_1 of individuals by the start of the next wave, whereas if it followed a pandemic wave (ie it was induced by the *new* virus) we assumed it would wane in a proportion Φ_2 of individuals. To link the proportion reporting symptoms to the other parameters, for each wave in each population, we used the final size equation (see main paper) based on the deterministic model and homogeneous mixing within each population.

Updating between waves: Between waves, the value of Z, the proportion susceptible (non-immune) was updated over each subclass of individuals in each population (see Figure 1 in main paper). We denote the expected proportions of persons in the respective states, at the relevant times, by: p(S_1), p(E_1S_2) etc, where p(S_1) = Z = proportion of the population in class S_1 before wave one. E_1 is the expected proportion reporting symptoms in the first wave (from the final size equation); $A_1 = E_1(1-\alpha_1)/\alpha_1$ is the frequency of asymptomatic infections; Φ_1 is the proportion of immune people who become susceptible again before the next wave if their immunity antedated wave 1, and Φ_2 is the proportion becoming susceptible again before the next wave when their immunity resulted from exposure in wave 1 or wave 2.

We need to calculate Z_2 = the proportion susceptible immediately before wave 2. We note that:

$$Z_2 = p(E_1S_2) + p(A_1S_2) + p(U_1S_2) + p(R_1S_2)$$

$$p(E_1S_2) = E_1\Phi_2$$

$$p(A_1S_2) = A_1\Phi_2$$

$$p(U_1S_2) = p(U_1) = Z - E_1 - A_1$$

$$p(R_1S_2) = (1-Z)\,\Phi_1$$

so it follows that

$$Z_2 = E_1\Phi_2 + A_1\Phi_2 + Z - E_1 - A_1 + (1-Z)\,\Phi_1$$

The details of the updating algorithm over three waves were complex, with multiple pathways because of the need to keep track of asymptomatic infections and to

distinguish the different states of immunity. For example, the true status of a person who is *apparently unaffected* in a particular wave could be any one of: *unexposed* (susceptible eg U_1 or non-susceptible); *exposed and immunised but not reporting clinical symptoms (eg A_1R)*; furthermore, *reported status could differ from the true status.* The full algorithm, implemented in MATLAB, used to track these multiple pathways can be made available on request.

Likelihoods of the observations in each population given the parameters were calculated to take account of the time sequence of waves and the directionality of infection, immune memory and loss. Each likelihood was based on the product of conditionally independent probabilities (each determined by current parameter values) for the seven sequential and parallel binary processes giving rise to the 2x2x2 matrix of outcomes (see Figure 2 in main paper). For example, for each population of size N, we designated the number reporting symptoms of influenza in wave 1 as N_1, and the number not reporting symptoms in wave 1 as N_0. We calculated E_1, the expected proportion reporting symptoms, using the final size equation applied to that wave and the current parameter values. Similarly we calculated E_0, the expected proportion not reporting symptoms, and then calculated the first binomial probability as a function of N, N_1, N_0, E_1 & E_0:

$$\Pr(N_1, N_0 \mid N, \text{parameters}) = (N!/N_1!N_0!)(E_1)^{N_1}(E_0)^{N_0}.$$

In practice, the constant factorial terms were omitted, as only the expectations change with the parameter estimates. For the second wave, there were two binomial probabilities, according to whether or not symptoms were reported in wave 1, and for wave three, there were four binomial probabilities, depending on the combinations of reports in the two earlier waves. The net effect of this chain (Figure 2 in main paper) is that the parameter-dependent part of the probability reduces to a log-linear model. The time sequence of the waves affects the way that the wave expectations depend on parameters (including the important initial condition for prior immunity Z).

Bayesian framework: The parameters were estimated in a Bayesian inference framework [3] with hyper-parameters defining the distributions of each of the

parameters across the 12 subpopulations. This allowed us to pool the information gained from the 12 subpopulations, without assuming that their parameters were identical. There were seven parameters within the mechanistic model (ie Z, Φ_1, Φ_2, α_1, α_2, α_3 and R_0). Each was allowed to vary among the 12 subpopulations. However all 12 subpopulation parameters were assumed to derive from the same prior distribution, described by the hyperparameters.

Hyperparameters: For the parameters bounded by 0-1 (ie Z, Φ_1, Φ_2, α_1, α_2, α_3) we modelled the relationship between the hyperparameters and the parameters as six beta-binomial distributions, each with unique hyperparameters a and b, means $m = a/(a + b)$ and variances $m(1 - m)/(1 + a + b)$. For each parameter (θ say) distributed in 0-1, the beta binomial contributed an additional factor to the likelihood as follows:

$$\Pr(\theta \mid a,b) = \left(\frac{\Gamma(a+b)}{\Gamma(a)\Gamma(b)} \right) \theta^{a-1}(1-\theta)^{b-1} .$$

For R_0, bounded below by 1, we used the gamma distribution for $R_0 - 1$, with mean a/b and variance a/b^2. Our belief in an acceptable range for R_0 was expressed in a factor incorporated into the prior for the mean R_0; we assumed that the factor was uniform in the range 1-8, and decreasing as exp(-(R_0-8)) for values in excess of 8. The corresponding prior probability distribution is proportional to:

$$\Pr(R_0 - 1 \mid a,b) \propto \begin{cases} 0 & \text{for } R0 \le 1 \\ b^a (R_0 - 1)^{a-1} \exp(-b(R_0 - 1)) / \Gamma(a) & \text{for } R_0 \le 8 \\ \exp(-(R_0 - 8)) b^a (R_0 - 1)^{a-1} \exp(-b(R_0 - 1)) / \Gamma(a) & \text{for } R_0 > 8 \end{cases}$$

.

The rationale for this choice of prior was as follows:

(1) In our previous work (supplementary information to [2]), using time dependent data from an outbreak of influenza in Saffron Walden Boarding school, the R_0 credibility interval included 8, so that value is not incredible.

(2) If $R_0 = 8$, and $Z = 0.5$, then the effective R is 4, and there is very little increase in attack rate for further increases in R_0 as the final size equation curve is almost flat. Without timing information, the data will not be able to discriminate different values of R above approximately 4.

(3) In a host population with typical mixing characteristics, the attack-rate from an influenza virus would thus be maximised above an R of 4, (eg R_0 of 8) although there could still be a selective advantage, in terms of rate-of-spread, for a virus with higher R_0.

In turn the hyperparameters had hyper-prior distributions

Pr(a)~U[0, 6]
Pr(b)~U[0, 6]

The joint posterior probability density of the full set of parameters and hyperparameters is proportional to:

Pr(hyperparms, params|obs) $\propto$ Pr(obs|params) Pr(params|hyperparams) Pr (hyperparams)

The marginal distributions of parameters and hyperparameters were derived through Bayesian integration methods described below.

MCMC simulation: The simulation generated individual parameter distributions for each population, as well as distributions of each hyperparameter. For each parameter, initial values were chosen from the prior distributions. We proposed a new value, θ'_k, from the current value using a symmetrical proposal distribution, a simple random walk with steps taken from the Uniform [-0.006 0.006] distribution, for Z, Φ_1, Φ_2, α_1, α_2, α_3 and the Uniform [-.06 0.06] for R_0. The metropolis acceptance equation for proposed θ'_k, was given by

$$\alpha = \min\left\{1, \frac{\Pr(\theta'_k \mid a_k, b_k)\Pr(y \mid \theta')}{\Pr(\theta_k \mid a_k, b_k)\Pr(y \mid \theta)}\right\}.$$

Each parameter was updated in turn. The hyperparameters were updated using a symmetrical proposal distribution, a simple random walk with steps taken from the uniform [-0.06 0.06] distribution and the acceptance probability was given by

$$\alpha = \min\left\{1, \frac{\Pr(\theta_k \mid a_k^{'}, b_k)\Pr(a')}{\Pr(\theta_k \mid a_k, b_k)\Pr(a)}\right\}.$$

Assessment of MCMC convergence: Convergence was assessed by visual inspection of multiple chains starting from widely different initial values, and by comparing the within *(W)* and between *(B)* chain variances for parameter estimates (*θ)* using Gelman & Rubin's multiple sequence diagnostic (3,4).

$$\hat{R} = \sqrt{\frac{\hat{V}(\theta)}{W}}$$

where $\hat{V}(\theta) = (1 - 1/n)W + (1/n)B$ and n is the chain length

Model deviance & complexity: [3,4,5] The posterior mean deviance after burn-in was calculated as the expectation of - 2 log likelihood. The complexity of each model after burn-in was calculated as

p_D = Posterior mean deviance – Deviance at particular parameter estimates

When we used maximum a posteriori (MAP) parameter estimates, the complexity estimate p_D was positive, whereas when median or mean parameter estimates were used, p_D estimates were always negative, although the negativity was less extreme for medians than for means. This finding implies that although the median and mean parameter estimates were representative of the central tendency for each parameter, the vector of medians (or means) was not representative, in large part because of the covariances between parameter estimates.

Accordingly, we calculated the alternative measure of complexity [4,5] as

p_V = Var (Deviance)/2

p_V is always positive, and it appeared to be a consistent measure of the effective number of fitted parameters in different model fits. We also noted that p_V was approximately twice the value of p_D obtained with the maximum likelihood parameter estimates. The Bayesian Deviance Information Criterion (DIC) was calculated from this complexity measure and the posterior mean deviance to provide an overall measure of fit [3,4,5] .

DIC = Posterior mean deviance + p_V.

Convergence: Figure S1 shows posterior parameter distributions, derived from the hyperparameter distributions, for the two independent chains generated for the 12 population model. Despite very different starting estimates, the chains give almost identical results (see also Tables S3 & S4). The Gelman & Rubin statistics (Table S1) provide more quantitative evidence of adequate convergence.

Comparison of models: Table S.2 compares the DIC estimates, based on p_V as the measure of complexity, for the results shown in Table 2 of the main paper. When the 8 urban populations and the 4 "school" populations were fitted in separate models, the deviance of the combined fit was 63 less than when the 12 populations were fitted in a single model. Although this improvement was at the expense of extra complexity (some 37 additional parameters), it was unlikely to be due to chance (probability of a χ^2 of 63 or more on 37 df is 0.0049). This reinforces the conclusion in the main paper that the "school" populations behave somewhat differently (See also Table 4 in main paper and Tables S3 & S4). Nevertheless, the 12 population results provide a convenient summary, and all practical conclusions from the two sets of results are equivalent.

Adequacy of model fits: Table 3 shows the actual fit for the 12 population model at the MAP for all parameters (including the hyper-parameters). For most populations the model clearly reproduces the attack-rates and the patterns of repeat attacks, as judged by the similarity of observed and expected numbers. The fit was somewhat less adequate for three of the "school" populations, and the results for Finchley School

tended to resemble those for the eight “urban” populations. As expected in such a non-linear model with correlated parameters, the fits using the vector of parameter median values were less adequate, and the fits using parameter means were quite poor.

Posterior parameter estimates: Table S3 presents posterior medians of parameters for each of the 12 populations from two independent simulations using all the populations, as well as the overall medians and confidence intervals derived from the hyperparameter distributions. The consistency of the results indicates that the simulation has adequately explored the plausible parameter space.

Table S4 presents median values and credibility intervals for effective R and also for R_0 by population in simulations involving all 12 populations, and in the subsets of 8 urban and 4 “school” populations.

SUPPORTING TEXT

Social Distancing

Intermittent social distancing could also contribute to wave-like behaviour of influenza, although it cannot, by itself, explain the pattern of repeated attacks from wave to wave. The simplest form of social distancing would be when susceptible persons protect themselves from exposure at a rate that is proportional to the number of symptomatic persons about them, which would tend to bring an outbreak to an end with fewer people attacked than would otherwise be the case. This assumption leads to a modified final size equation:

$$E = \left(\frac{\alpha Z}{1 + \alpha\Delta / R_0} \right) \left(1 - e^{-((R_0/\alpha) + \Delta)E} \right).$$

The new parameter Δ measures the strength of social distancing. This equation is equivalent to the final size equation in the main paper, with the transformation:

$$R_0 = R_0' - \alpha\Delta.$$

So if this simple form of social distancing were present, we could not detect it unless we had some independent means to estimate Δ and re-interpret R_0. However, a reassuring corollary of this form of social distancing is that it does not, of itself, invalidate the assumption of homogeneous mixing. In further work using mortality data from the three waves of the 1918-19 pandemic in England and Wales, we will try to estimate Δ by assuming that the propensity for people to distance themselves from exposure increases with mortality rate, which differed from wave to wave.

Demarcation of waves and waning of resistance

It has been suggested that if the three waves in 1918-19 overlapped, this might have invalidated our use of the final-size equation. Inspection of parallel mortality data [1] shows clear demarcation between the three waves, although we do not have week by week counts for the morbidity data used in our study. Because we used lumped data (in waves), our analysis of "waning resistance" was likewise based on lumping ("proportion waned") rather than a quantitative rate of waning.

It would have been ideal to allow "waning resistance" to vary with wave, as well as with exposure history. However, to minimise complexity, we introduced a parameter, Φ_1, for waning of the resistance that antedated wave 1 (arguably due to prior exposure to seasonal influenza), and a separate parameter for waning of resistance acquired from exposure to the new virus, Φ_2, acquired following exposure in wave 1 or wave 2. Neither parameter was assumed to vary systematically from interval (wave 1-wave 2) to interval (wave 2- wave 3). However, as the latter interval was slightly longer than the former in real time, our "proportion waned" method corresponds to an implicit "between interval" adjustment of the relevant rate parameters in the expected direction, and this is consistent with the parameter estimates from our model, where "waning" seems more pronounced in the school populations.

We have not attempted to formally partition the "waning of resistance" between loss of immunity and antigenic drift. However, the waning of resistance was greater for younger (school) populations than for adult populations, suggesting that waning immunity may have been more important than antigenic drift. This is supported by

collateral evidence that in childhood, it can take at least several exposures to seasonal influenza to build up a solid base of immunity.

Incremental nature of acquired immunity

The model fit with $\Phi_2 = 0$ was poor, indicating that we can exclude the possibility that acquired immunity was permanent after first exposure to the pandemic virus.

Possibility of virus variation between waves

We have not presented results that allow for strain (and hence possibly R_0) differences between waves. However, there is no definitive evidence of strain variation from wave to wave, as only a single genetic sequence has been recovered from the UK material in the 1918-19 pandemic. All three waves had the “pandemic signature” of increased mortality in younger adults. Some commentators would suggest that the higher mortality in waves 2 & 3 was due to a genetic increase in virulence; this may be so, but other explanations are also possible. Even if there were a genetic change in virulence from wave to wave, this would not necessarily translate into changes in R_0.

Our assumption that R_0 is the same from wave to wave for any particular population, is unlikely to be strictly true. However, as R_0 depends on the mixing characteristics of each population, as well as the nature of the virus, we believe that our approximating assumption can be justified, particularly if there were little genetic change in the virus. Indeed, in other (unpublished) analyses we allowed R_0 to vary systematically from wave to wave, with no significant improvement in fit. This implies that much of the variation in R_0 was due to population characteristics rather than to the virus. (Of course the virus had additional effects on R through its effects on population immunity.) For reasons of simplicity we have therefore reported the results based on an R_0 that can vary from population to population but which does not vary systematically from wave to wave.

It has also been suggested that we cannot actually estimate R_0 from our data, but only R, which depends on both R_0 and the proportion susceptible (which we designate as Z). However, the whole point of our paper is that we have derived estimates of both R_0 and Z for each population, and their distributions between populations, and thus estimated the distributions of initial $R = R_0.Z$ that best fit the data. Our method has

probably succeeded because the data give wave-to-wave attack rates for individuals, with the 2x2x2 data matrix implicitly providing information on susceptibility and immunity, and thus allowing us to separate the effects of R_0 from those of Z (the proportion susceptible).

Possibility of seasonal changes in bacterial infection

It has also been suggested that seasonal changes in bacterial infection might compensate for the loss of susceptibility to influenza, and by implication, that this could weaken our model. We agree that bacterial infection is one of many factors that, in the interests of parsimony, we have not explicitly incorporated in our model. However, we note that by allowing the proportion of infections causing symptoms to vary from wave to wave, through α_1, α_2 and α_3, and to vary from population to population, through the hyperparameters, our model is flexible enough to accommodate effects of factors such as bacterial infection that are not explicit in the model.

Comments on mortality

We agree that the age distribution of mortality in 1918-19, relatively higher in young adults, is of particular interest. It suggests that older individuals may have had protection from previous exposure to a virus, antigenically related to H1N1, circulating in the years prior to 1890. Younger people may have been protected by innate or cross-reactive immunity. However, in this paper, we have analysed morbidity data (symptoms) rather than mortality, and the immune mechanisms protecting against mortality and morbidity may not be identical.

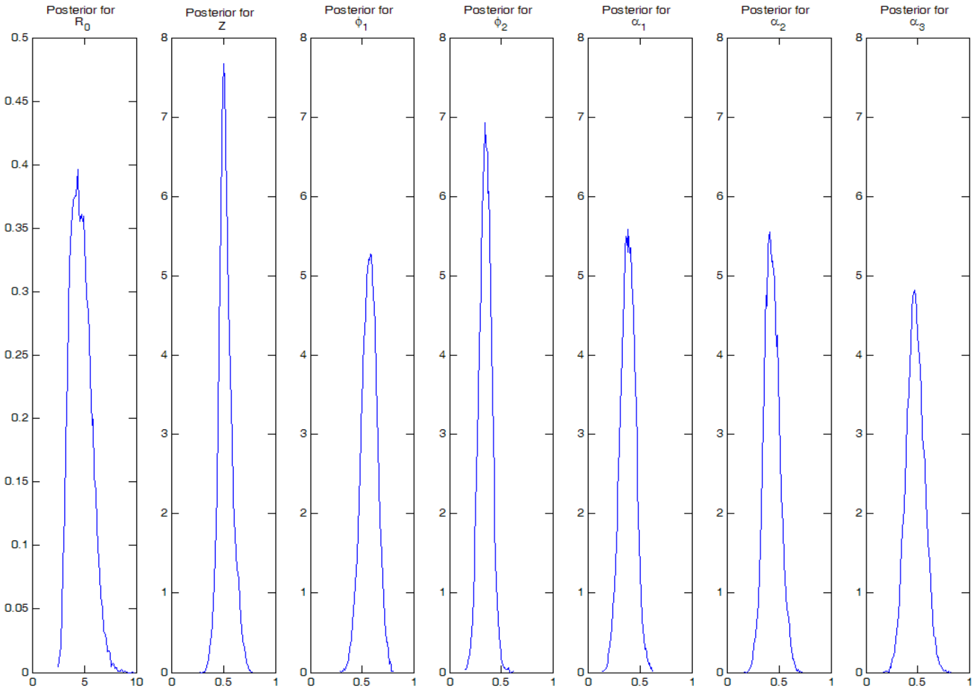
Posterior for R0
Posterior for Z
Posterior for φ1
Posterior for φ2
Posterior for α1
Posterior for α2
Posterior for α3

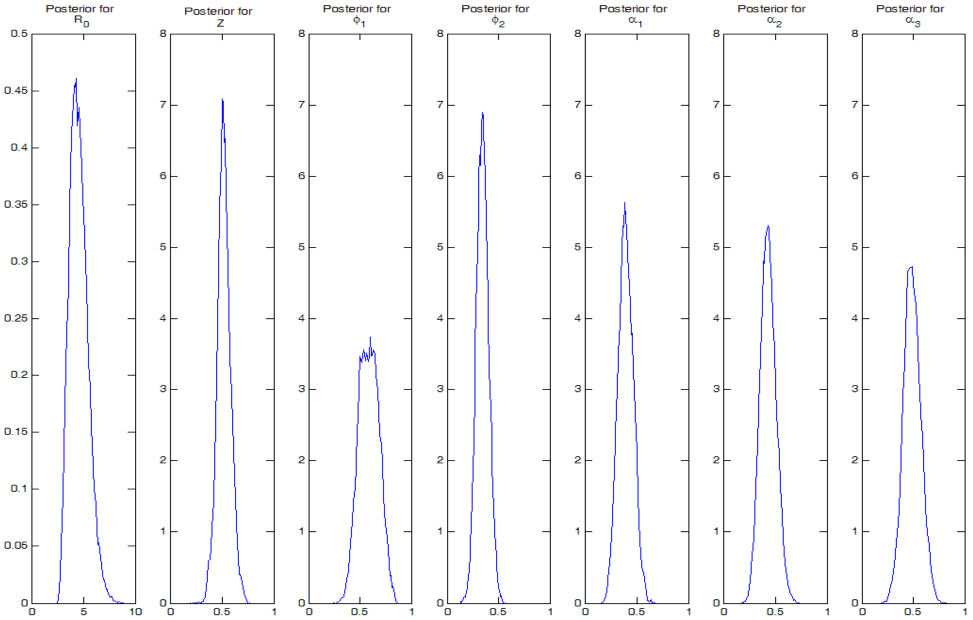
Posterior for R0
Posterior for Z
Posterior for φ1
Posterior for φ2
Posterior for α1
Posterior for α2
Posterior for α3

Legend to Figure S1.1 & Figure S1.2

Posterior parameter distributions from MCMC simulation of the 12 population model, derived from the joint distributions of the hyperparameters. Figure S1.1 shows the results for 1 in 10 trimmed output for simulation 1 (294,000 iterations after burn-in over all parameters and hyperparameters) and Figure S1.2 shows the analogous results for simulation 2 (320,000 iterations after burn-in). It can be seen that the derived parameter distributions from the separate chains of simulations have given very similar results, and as shown in Table S1, there is objective evidence that the two chains have converged towards the same solution space. Such posterior estimates give an overall summary of how parameter differences can explain the range of attack-rates and re-attack-rates in different populations in 1918-19.

Table S1

Adequacy of convergence of the 12 population model as judged by the Gelman & Rubin statistic ($\hat{R}$) comparing two independent chains

**Parameter**	R_0	Z	Φ_1	Φ_2	α_1	α_2	α_3
$\hat{R}$ statistic	1.031	1.000	1.004	1.002	1.017	1.000	1.021

Note for Table S1

The Gelman and Rubin statistic assesses convergence by comparing the within and between chain variance for parameter estimates. Values of $\hat{R}$ less than 1.1 provide evidence of adequate convergence [3,4]. These results, based on parameter estimates derived from the joint hyperparameter distributions, suggest that both simulation chains show adequate convergence.

TABLE S2

Comparison of models fits shown in Table 2 of main paper

**Model**	**Deviance**	**Complexity (p_v)**	**DIC**
12 populations fitted jointly	47266	90	47356
8 urban populations	35824	68	35892
4 "school" populations	11379	59	11438
Urban and "school" population models fitted separately	47203 (-63)	127 (37)	47330 (-26)

Note to Table S2

When the 8 urban populations and the 4 "school" populations were fitted in separate models, the deviance of the combined fit was 63 less than when the 12 populations were fitted in a single model (simulation 1; results with simulation 2 were virtually identical). Although this improvement was at the expense of extra complexity (some 37 additional parameters), it was unlikely to be due to chance (probability of a χ^2 of 63 or more on 37 df is 0.0049). This reinforces the conclusion in the main paper that the "school" populations behave somewhat differently (See also Table 2 in main paper and Tables S3 & S4). Nevertheless, the 12 population results provide a convenient summary that is "good enough" for many purposes.

TABLE S3

POPULATION-SPECIFIC MEDIAN PARAMETER ESTIMATES

Population	R_0	Z	Φ_1	Φ_2	α_1	α_2	α_3
**South Shields**	3.6628	0.4283	0.3272	0.4352	0.2177	0.1960	0.2874
	3.7515	0.3926	0.3286	0.4141	0.2283	0.2105	0.3199
**Leicester**	4.0303	0.3997	0.6359	0.2226	0.3295	0.2795	0.4300
	3.6977	0.3820	0.6309	0.2207	0.3687	0.2734	0.5286
**Wigan**	4.2480	0.4743	0.4566	0.1024	0.1506	0.3204	0.6400
	3.5790	0.4882	0.4022	0.1066	0.1629	0.3665	0.6868
**Newcastle**	5.0691	0.3941	0.7637	0.2610	0.2162	0.0863	0.6087
	5.2306	0.3645	0.7270	0.2672	0.2379	0.0883	0.5378
**Manchester**	4.9365	0.5276	0.4884	0.5198	0.3371	0.2189	0.0580
	4.3907	0.5153	0.5072	0.5525	0.3691	0.2019	0.0592
**Blackburn**	4.2770	0.5282	0.3839	0.4336	0.2196	0.2058	0.2276
	4.1890	0.5455	0.4921	0.4855	0.2221	0.1677	0.2238
**Widnes**	3.4855	0.5885	0.4638	0.1294	0.2906	0.5240	0.5605
	3.7065	0.6057	0.5051	0.1222	0.2590	0.5393	0.5639
**London police**	3.8834	0.4566	0.6043	0.3194	0.2955	0.3303	0.1367
	3.5564	0.4455	0.5570	0.2685	0.3355	0.3681	0.1683
**Cambridge Uni**	5.1445	0.5282	0.6922	0.1841	0.5298	0.6337	0.4974
	5.6549	0.6002	0.7702	0.1739	0.4327	0.6629	0.5129
**Clifton College**	5.6566	0.6450	0.8536	0.4872	0.5805	0.4630	0.9586
	5.5655	0.6431	0.8658	0.4900	0.5796	0.4586	0.9544
**Haileybury**	4.9823	0.7616	0.4467	0.3228	0.4765	0.8504	0.8675
	4.8211	0.7995	0.4960	0.3290	0.4535	0.8168	0.8454
**Finchley School**	4.4010	0.4269	0.7421	0.2353	0.3664	0.6342	0.0951
	3.7096	0.4480	0.7024	0.2430	0.4177	0.6366	0.1047
**OVERALL (1)**	4.5893	0.5150	0.5716	0.3484	0.3874	0.4299	0.4759
	(3.0953-6.7415)	(0.4083-0.6639)	(0.4172-0.7112)	(0.2333-0.4626)	(0.2452-0.5215)	(0.2961-0.5858)	(0.3144-0.6485)
**OVERALL (2)**	4.4878	0.5188	0.5859	0.3438	0.3914	0.4304	0.4866
	(3.1007-6.5323)	(0.3922-0.6535)	(0.4029-0.7707)	(0.2329-0.4651)	(0.2547-0.5384)	(0.2919-0.5896)	(0.3253-0.6587)

Notes to Table S3

The first population-specific median in each cell is derived from simulation 1, and second from simulation 2 of the 12 population model. The "overall" medians and 95% credibility intervals are estimated from the hyperparameter distributions in simulation 1 and simulation 2 respectively.

TABLE S4

Median values and 95% credibility intervals for effective *R* and R_0 for each population in different simulations

Population	*R*	*R*	*R*	*R*	R_0	R_0	R_0	R_0
**Simulation**	**1**	**2**	**3a**	**3b**	**1**	**2**	**3a**	**3b**
**South Shields**	1.30 (1.07-4.87)	1.31 (1.06-4.52)	1.19 (1.06-3.17)		3.66 (1.75-7.81)	3.75 (2.13-7.64)	3.59 (1.83-7.56)	
**Leicester**	1.51 (1.11-3.01)	1.41 (1.16-2.82)	1.41 (1.14-2.96)		4.03 (1.88-7.82)	3.70 (2.41-7.55)	3.76 (2.32-7.07)	
**Wigan**	1.62 (1.16-5.40)	1.48 (1.14-4.25)	1.62 (1.13-3.81)		4.25 (2.06-8.16)	3.58 (1.81-7.35)	3.48 (1.80-6.89)	
**Newcastle**	1.90 (1.29-3.50)	1.83 (1.25-3.44)	1.44 (1.19-3.28)		5.07 (2.46-8.29)	5.23 (3.37-8.49)	4.62 (2.29-7.94)	
**Manchester**	2.49 (1.31-6.77)	2.12 (1.41-4.32)	1.63 (1.23-3.84)		4.94 (2.66-8.58)	4.39 (2.32-8.19)	3.46 (2.02-7.24)	
**Blackburn**	2.12 (1.25-5.96)	1.93 (1.16-5.52)	1.61 (1.15-4.13)		4.28 (2.06-8.06)	4.19 (1.78-7.85)	3.16 (1.77-6.94)	
**Widnes**	1.93 (1.25-4.28)	2.12 (1.36-4.79)	1.88 (1.31-3.67)		3.49 (1.86-7.49)	3.71 (2.07-7.52)	3.42 (1.94-6.55)	
**London police**	1.52 (1.08-4.05)	1.41 (1.10-3.71)	1.39 (1.13-3.12)		3.88 (1.74-7.81)	3.56 (1.79-7.80)	3.57 (2.07-7.31)	
**Cambridge Uni**	2.60 (1.51-5.09)	3.10 (1.76-5.41)		3.44 (1.38-6.82)	5.14 (2.66-8.58)	5.65 (3.16-8.78)		5.57 (2.07-9.22)
**Clifton College**	3.55 (2.14-5.58)	3.51 (2.16-5.75)		3.67 (1.96-6.27)	5.66 (3.09-8.63)	5.57 (2.97-8.71)		5.63 (2.68-9.19)
**Haileybury**	3.67 (2.20-6.26)	3.70 (2.01-6.18)		3.87 (1.86-6.51)	4.98 (2.82-7.97)	4.82 (2.34-7.75)		4.91 (2.14-8.07)
**Finchley School**	1.83 (1.26-3.66)	1.58 (1.21-3.27)		1.35 (1.15-4.02)	4.40 (2.49-8.31)	3.71 (2.17-7.59)		2.92 (1.75-7.94)
**OVERALL**	2.33 (1.57-3.96)	2.32 (1.60-3.36)	1.81 (1.16-2.94)	2.84 (1.43-5.36)	4.59 (3.10-6.74)	4.49 (3.10-6.53)	3.80 (2.53-5.92)	4.73 (2.43-8.42)

Notes to Table S4.

For each population, credibility intervals for parameters were estimated from (joint) distributions of population-specific simulations, conditional on the hyperparameter distributions. Overall credibility intervals were derived from the (joint) distributions of the hyperparameters. Results from simulation 1 are based on 294,000 iterations, after burn-in, over all 12 populations. Results from simulation 2 are based on 320,000 iterations, after burn-in, over all 12 populations, with entirely different starting estimates for the parameters. Results from simulation 3a are based on 480,000 iterations, after burn-in, for the eight non-school populations, while results from simulation 3b are based on 1.2 million iterations after burn-in, for the four "school" populations.

SUPPORTING REFERENCES

1. Ministry of Health. *Pandemic of Influenza 1918-1919. in Reports on Public Health and Medical Subjects* Vol. 4 (ed. HMSO) (Ministry of Health, London, England, 1920).
2. J.D. Mathews, C.T. McCaw, J. McVernon, E.S. McBryde, J.M. McCaw, *PLoS ONE* **2**, e1220 (2007).
3. J. Gill, *Bayesian methods : a social and behavioral sciences approach*. Chapman & Hall/CRC. (2008).
4. D.J. Spiegelhalter, N.G. Best, B.P. Carlin, A. Van der Linde. *J. R. Statist Soc B 64(4) 583-639 (2002)*
5. Spiegelhalter DJ. Some DIC slides. MRC Biostatistics Unit. (2006) *www.mrc-bsu.cam.ac.uk/bugs/winbugs/**DIC-slides**.pdf*
